# Metabolic Profiling in Bipolar Disorder Patients During Depressive Episodes

**DOI:** 10.3389/fpsyt.2020.569612

**Published:** 2020-12-16

**Authors:** Yan Ren, Shuang Bao, Yuan Jia, Xiao-li Sun, Xiang-xin Cao, Xiao-ying Bai, Jun-Sheng Tian, Hong Yang

**Affiliations:** ^1^Department of Psychiatry, Shanxi Bethune Hospital, Shanxi Academy of Medical Science, Taiyuan, China; ^2^Biotherapy Lab, Shanxi Bethune Hospital, Taiyuan, China; ^3^Modern Research Center for Traditional Chinese Medicine of Shanxi University, Taiyuan, China

**Keywords:** bipolar disorder, bipolar depression, metabonomics, nuclear magnetic resonance, biomarker

## Abstract

Bipolar disorder (BD) is a common and debilitating mental disorder. Bipolar depression is the main episode of BD. Furthermore, there are no objective biomarkers available for diagnosing the disorder. In this research, a Nuclear Magnetic Resonance (NMR) spectroscopy based on a metabonomics technique was used to analyze serum samples from 37 patients with bipolar depression and 48 healthy control participants to determine potential biomarkers for bipolar depression. In total, seven different metabolites were identified that could effectively distinguish patients from healthy controls. The metabolites indicated that disturbances of amino acid and energy metabolisms might be involved in the pathogenesis of BD. Finally, a panel consisting of four potential biomarkers (lactate, trimethylamine oxide, N-acetyl glycoprotein, and α-glucose) was identified, which showed a higher combined diagnostic ability with an area under the curve of 0.893. Our findings may contribute to the development of an objective method for diagnosing bipolar depression.

## Introduction

Bipolar disorder (BD) is a common and debilitating mental disorder, characterized by mood disturbances that comprise periods of mania, depression, and euthymia, and the lifetime prevalence of BD is about 5.5–7.8% in the general population (1). A variety of genetic, biochemical, and environmental factors seem to be involved in the pathogenesis of BD. Currently, however, the diagnosis only depends on the subjective recognition of clinical symptoms, without any support from objective methods (2). This fact may lead to inadequate treatments and outcomes. Bipolar depression is the main episode of BD, which occurs more frequently than either hypomania or mania (3). To obviate this situation, it is vital to identify objective biomarkers for bipolar depression.

Metabonomics mainly investigates the changes of small molecule metabolites in organisms, providing the potential to reveal the pathogenesis of a disease and to identify objective biomarkers (4). It is a new research method following genomics, transcriptome, and proteomics. In recent years, metabonomics has been extensively applied to characterize the metabolic signature of neuropsychiatric disorders, such as autism, stroke, Parkinson's disease, multiple sclerosis, and schizophrenia (5–10). NMR spectroscopy is one of three major analytical techniques used in metabonomic mapping. It has the benefits of speedy analysis, is non-invasive, and provides a comprehensive coverage of metabolome, making it a common method of metabolomics (11, 12).

Previous studies identified a panel of metabolic biomarkers that could discriminate patients with BD from healthy controls (13–16, 16–26). Lan et al. (14) found several differential metabolites in post-mortem brain tissue, indicating that the balance of excitatory/inhibitory neurotransmission is the core of BD. Yoshimi et al. (13) reported a metabonomics analysis of cerebrospinal fluid samples from BD patients and controls. The results indicated that isocitric acid played a key role in the onset of BD. Other studies has also identified some potential biomarkers for BD diagnosis both in blood and urine (15, 16, 16–26). However, the patients included in these studies were in different episodes. One study took this problem into consideration, but only a urine sample was used. In this study, a panel consisting of five differential metabolites was identified (isobutyric acid, formic acid, 2,4-dihydroxypyrimidine, azelaic acid, and sucrose) (26).

In this study, an NMR-based metabonomics approach was applied to identify metabolic alterations in the plasma of patients with BD during depressive episodes. Our intention was to identify potential biomarkers for BD diagnosis. We also aimed to achieve a better understanding of the pathways affected in BD.

## Materials and Methods

### Subject Recruitment

The protocols of this study were reviewed and approved by the medical ethics committee of Shanxi Bethune Hospital. All subjects and their guardians voluntarily participated in the study and gave informed consent. For the teenage participants, informed consent was signed by their parents. Experienced and licensed psychiatrists participated in the recruiting procedure. Thirty-seven patients with BD were recruited from the psychiatry department of Shanxi Bethune Hospital, including 23 patients who were receiving medication. All individuals in the BD group, between the ages of 15 and 50, fulfilled bipolar depression criteria of the DSM-5 (Diagnostic and Statistical Manual of Mental Disorders, Fifth Edition). Patients with any physical or other mental disorders were excluded, as were patients who had substance abuse issues. A control group of 48 healthy participants was recruited from the medical examination center of the same hospital. To be eligible for the healthy control group, volunteers needed to not have any psychiatric or mental disorders, substance abuse, and the family of the volunteers also needed to have no history of major mental disorders.

### Sample Collection

The blood samples of all subjects were taken by professionals at the Shanxi Bethune Hospital in the morning after 12 h of fasting. The blood was mixed upside down after clotting and kept for half an hour at room temperature before being centrifuged at 3,000 r/min for 15 min. Then the supernatant (plasma) was drawn with a pipette, evenly transferred into clean Eppendorf tubes, and stored at 80°C for later analysis.

### NMR Acquisition

The procedure of NMR analysis was as follow: (1) 450 μL plasma samples were thawed and mixed with 350 μL of deuterated water; (2) The mixture was centrifuged at 13,000 r/min for 20 min; (3) 600 mL of the supernatants transferred into 5 mm NMR tubes; (4) ^1^H-NMR spectra of the plasma samples were collected on a Bruker 600-MHz AVANCE III NMR spectrometer, using one-dimensional Carr-Purcell-Meibom-Gill NMR spectra (27).

### Data Processing

The baseline and phase pretreatment of ^1^H NMR spectra were performed by MestReNova software (Mestrelab Research, Santiago de Compostella, Spain). Chemical shift correction was carried out based on creatinine at δ 3.04 ppm. In the unit of δ 0.01 ppm, the spectra were segmented across the region of δ 0.50–5.50 ppm. The region of δ 4.70–5.20 ppm was excluded due to residual water. The data were then normalized to the total sum of the spectra.

### Statistical Analysis

Spectra data were imported into SIMCA-P 14.1 software for building an Orthogonal Partial Least Square Discriminant Analysis (OPLS-DA) model. This model was used to visualize the discrimination between the different groups. The quality of the model was evaluated by the results of the permutation test (200 cycles) and the values of *R*^2^ and *Q*^2^. To discover the differential metabolites contributing to samples separation, the loading plot of the OPLS-DA model was established and analyzed. A number of metabolites responsible for samples differentiation could be identified based on the variable importance in the project (VIP) threshold of 1. The diagnostic ability of potential biomarkers was evaluated by The Receiver Operating Characteristic Curve (ROC) with the area under the curve (AUC). Meanwhile, to discover the interaction of differential metabolites, the metabolites were imported into online software MetaboAnalyst for pathway analysis.

## Results

### Clinical Characteristics

A total of 37 patients and 48 healthy control participants were included in the research. Their detailed information is shown in Table 1. There were no significant differences in gender or age between the patient and control groups (*P* > 0.05), indicating that the findings cannot be attributed to demographic factors. There were two patients with bipolar I (at least one manic episode with or without major depressive and hypomanic episodes) and 34 patients with bipolar II (one or more major depressive and hypomanic episodes without manic episode). We found that the diagnostic age of all patients was later than the onset age, indicating the failure of timely diagnosis and treatment. We also found more depressive episodes or mixed episodes than manic episodes. Furthermore, there were significant depression and anxiety symptoms in the bipolar depression group.

**Table 1:** Demographic and clinical details of recruited subjects.

**Variables**	**Patients**	**Healthy controls**	***p*-value**
Sample size	37	48	—
Age (year)	32.03 ± 13.15	30.37 ± 7.13	0.461
BMI	24.77 ± 4.32	23.49 ± 2.80	0.103
Gender (male/female)	16/21	23/25	0.668
**Diagnosis**			
BD type I	2 (5.4%)	—	—
BD type II	34 (91.9%)	—	—
BD uncertain	1 (2.7%)	—	—
HAMD-24 total score	25.05 ± 5.56	—	—
**HAMD factor score**			
Anxiety/ Somatization	5.81 ± 2.38	—	—
Weight	0.43 ± 0.6	—	—
Cognitive impairment	4.43 ± 1.98	—	—
Diurnal variation	0.65 ± 0.82	—	—
Slowness	6.11 ± 1.93	—	—
Sleep disorder	2.78 ± 1.47	—	—
Despair sense	4.78 ± 1.95	—	—
Suicide	1.08 ± 0.86		
HAMA-14 total score	19.35 ± 5.85	—	—
**HAMA factor score**			
Somatic anxiety	6.97 ± 3.84	—	—
Mental anxiety	12.30 ± 3.57	—	—
CGI score	5 ± 0.62	—	—
Age of first onset (age)	27.2 ± 13.9	—	—
Total course of disease (month)	84.03 ± 90.4	—	—
Total incidents of emotional attack	7.56 ± 6.44	—	—
incidents of depression in the past	4 ± 4.95	—	—
incidents of hypomania in the past	4.81 ± 7.09	—	—
incidents of mania in the past	0.05 ± 0.23	—	—
incidents of mixed state in the past	0.61 ± 3.34	—	—
Diagnostic age (year)	33.9 ± 14.8	—	—
Medication (Yes/No)	23/14	—	—
**Medicine**			
Mood stabilizers	14 (37.8%)	—	—
Lithium	8 (21.6%)	—	—
Lamotrigine	4 (10.8%)	—	—
Sodium valproate	2 (5.4%)	—	—
Antipsychotics	14 (37.8%)	—	—
Antidepressants	18 (48.6%)	—	—

### Analysis of ^1^H NMR Profiles

**Figure 1:**
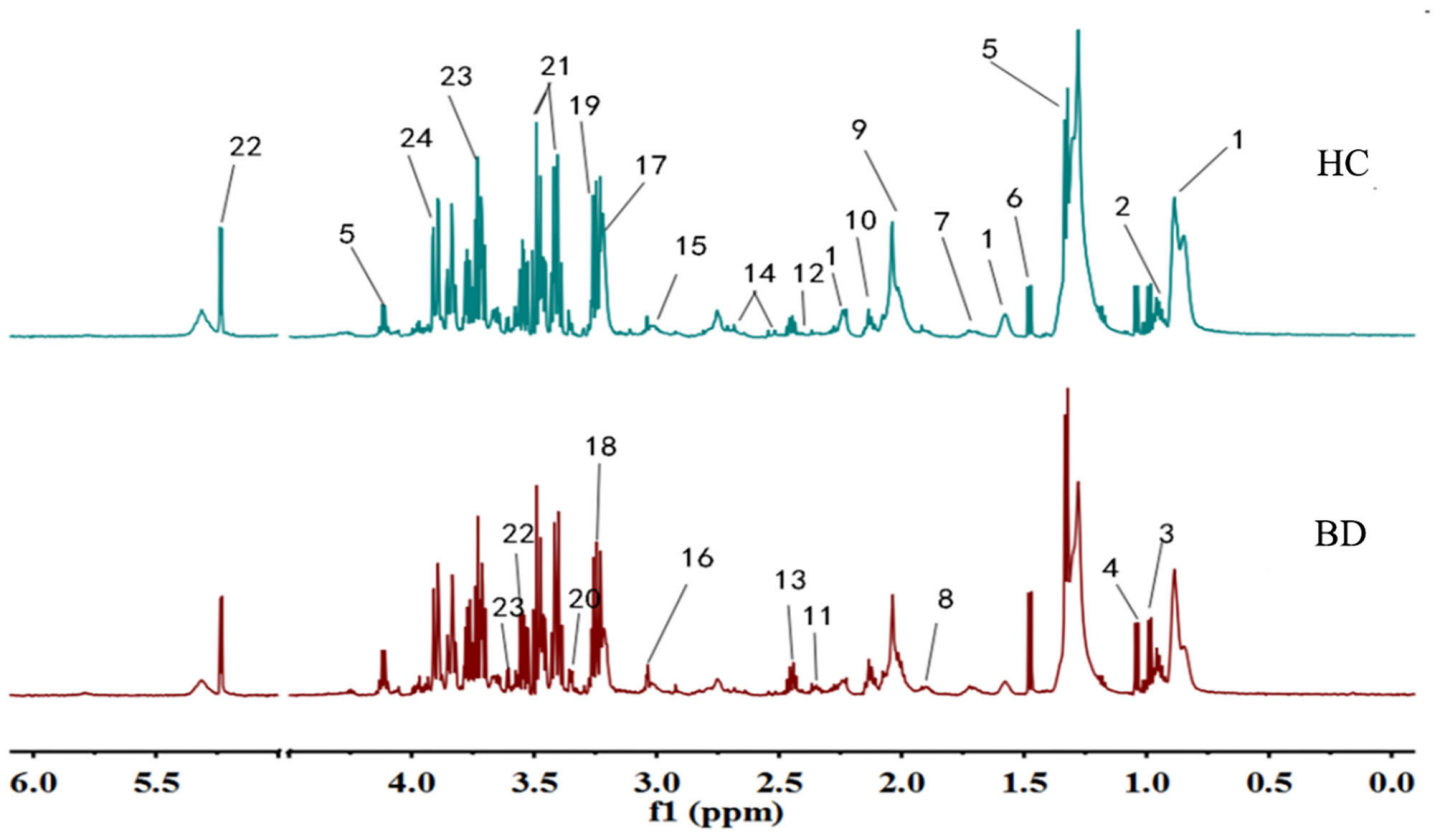
Typical IH NMR spectrum of plasma in patients and healthy controls. 1. Lipids; 2. Leucine; 3. Isoleucine; 4. Valine; 5. Lactic acid; 6. Alanine; 7. Lysine; 8. Acetate; 9. N-acetylglycoprotein; 10. Glutamine; 11. Beta-hydroxybutyric acid; 12. Pyruvic acid; 13. Glutamic acid; 14. Citric acid; 15. Creatine; 16. Creatinine; 17. Choline; 18. Trimethylamine Oxide; 19. Beta-Glucose; 20. Proline Acid; 21. Alpha glucose; 22. Glycine; 23. Threonine; 24. Aspartic acid.

Typical ^1^H NMR spectrum from plasma samples is shown in Figure 1. Referring to the human metabolome database (HMDB) (http://www.hmdb.ca/) and related reports over time, 24 compounds (13, 14) were identified in the plasma samples from the ^1^H NMR spectrum.

### OPLS-DA Model Recognition

**Figure 2:**
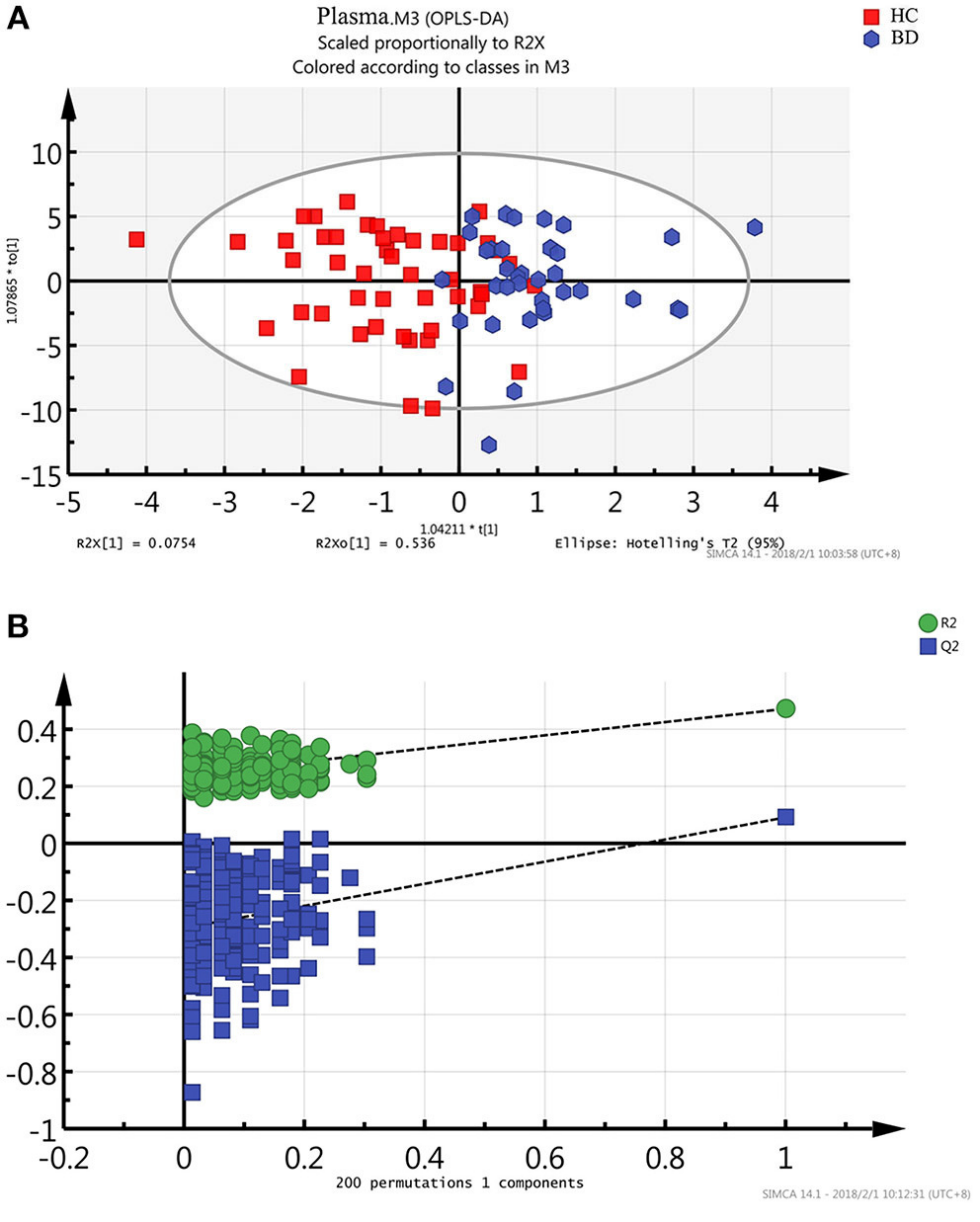
Metabonomic analysis of plasma Samples. **(A)** The score plot of the OPLAS-DA model showing a clear discrimination between BD subjects and healthy controls. **(B)** Statistical validation of the OPLS-DA model by permutation testing.

Metabonomics obtains a large amount of multi-dimensional information, and requires a series of statistical methods to gain effective information. Using the idea of orthogonal signal correction, Orthogonal Partial Least Squares Discriminant Analysis (OPLS-DA) can filter out some random noises, better distinguish between groups, and improve the validity and analytical ability of the model (see Figure 2A). The score plot of OPLAS-DA model showed a clear discrimination between patients with BD and healthy control participants, with little overlap. Permutation testing showed that the cumulative *R*^2^ and *Q*^2^ values were all less than the original value, indicating that the OPLS-DA model built in this study was not overfitting (Figure 2B).

### Candidate Biomarkers

**Figure 3:**
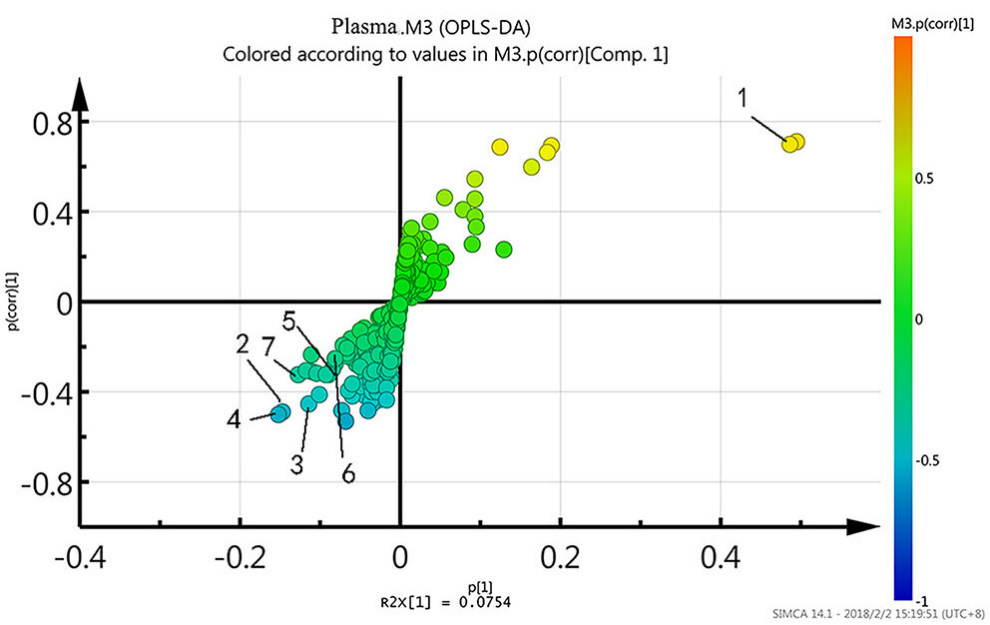
The loading plot of the OPLS-DA model.

To further identify differential metabolites responsible for samples separation, the loading plot (Figure 3) was constructed based on the OPLS-DA model. The farther away from the central origin, the greater the contribution to the separation of the bipolar depression group from the healthy control group, and the greater the likelihood of differential metabolites. VIP value was used as the evaluation index. The metabolites were screened by the criterion of VIP > 1, and the normalized peak area of metabolites was *t*-tested by SPSS software (*P* < 0.05). Finally, seven differential metabolites were obtained in plasma: lactate level increased significantly, while trimethylamine oxide, N-acetyl glycoprotein, choline, α-glucose, glycine, and β-glucose levels decreased significantly (Table 2).

**Table 2:** Differential metabolites in plasma of patients with bipolar depression (VIP > 1, *P* < 0.05).

**No**.	**Metabolites**	**Chemical shift**	**Normalized peak area (×100)**		***P*-value**	**VIP**
			**Controls**	**BD**		
1	Lactate	1.33	2.67 ± 0.65	3.57 ± 1.10	5.25918E-05	9.51
2	Trimethylamine oxide	3.24	0.67 ± 0.20	0.56 ± 0.14	0.005753086	2.98
3	N-acetyl glycoprotein	2.05	0.86 ± 0.14	0.79 ± 0.10	0.007256015	2.23
4	α-glucose	3.5	0.35 ± 0.2	0.27 ± 0.15	0.04239548	2.90
5	Choline	3.55	0.67 ± 0.15	0.60 ± 1.14	0.029009085	2.09
6	β-glucose	3.27	0.54 ± 0.15	0.46 ± 0.16	0.039868585	1.98
7	Glycine	3.21	0.74 ± 0.13	0.67 ± 0.14	0.018977458	1.23

### Pathway Analysis of Candidate Biomarkers

**Figure 4:**
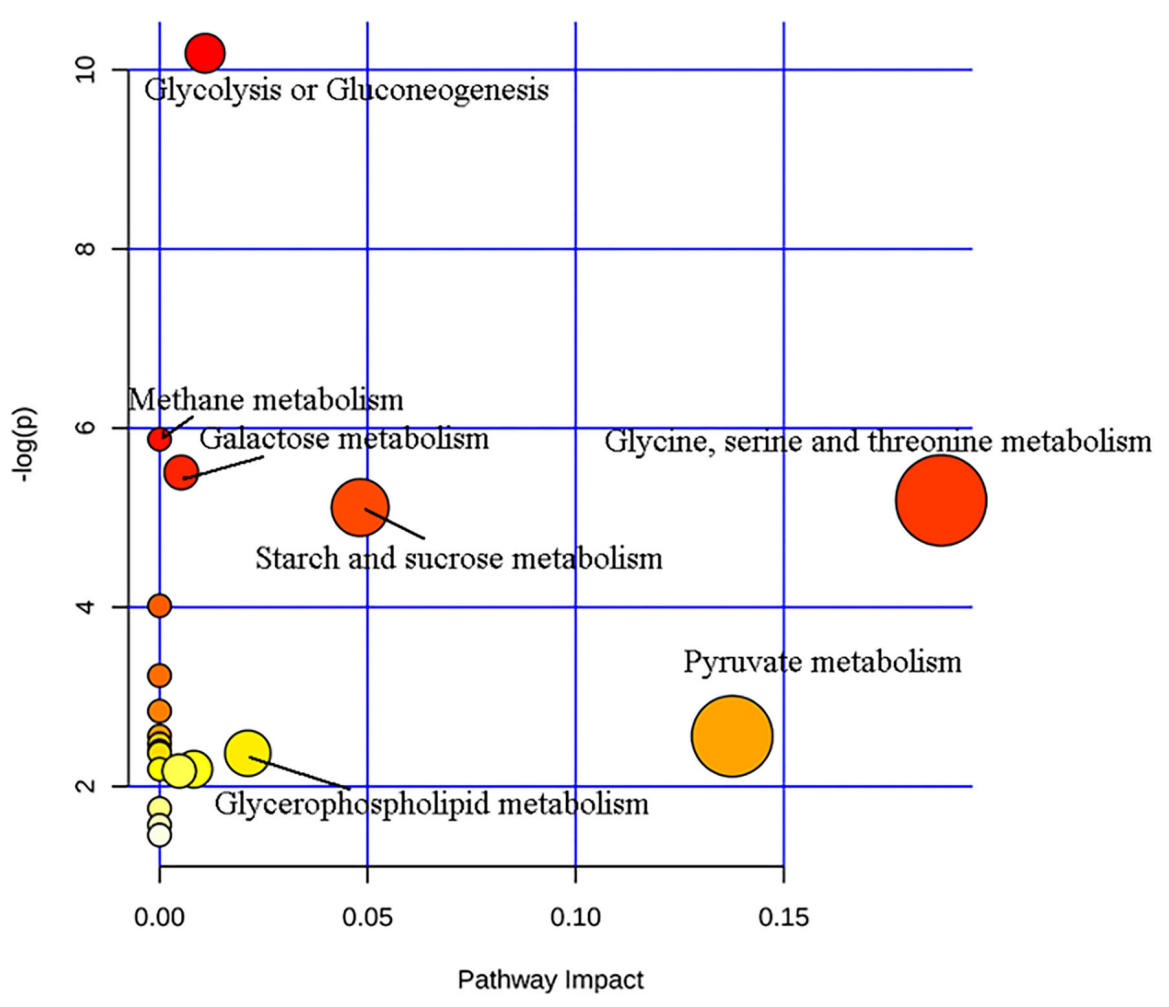
Metabolic pathways analysis of differential metabolites.

Through analysis of metabolic pathways, we found that the main metabolic pathways involved in differential metabolites were: (1) glycolysis or gluconeogenesis, (2) methane metabolism, (3) galactose metabolism, (4) glycine, serine and threonine metabolism, (5) starch and sucrose metabolism, (6) pyruvate metabolism, and (7) glycerophospholipid metabolism (Figure 4). Glycine, serine and threonine metabolism, and pyruvate metabolism showed a higher correlation with bipolar depression (pathway impact >0.1 and *P* < 0.05).

### Identification of Biomarkers and Evaluation of Diagnostic Ability

The ROC and the area under the curve (AUC) are performed to assess the diagnostic function of differential metabolites. The closer the AUC is to 1, the better diagnostic performance is demonstrated. AUC <0.5 explains no diagnostic function. In this study, ROC curves were also used to compare the diagnostic function of single candidate biomarkers in plasma. The results showed that most AUC values were <0.6, indicating a lower diagnostic function. Because of the robust interaction between endogenous metabolites, single metabolites could not predict a disease accurately, while using a combination of different metabolites has greater efficacy, improving diagnosis.

**Figure 5:**
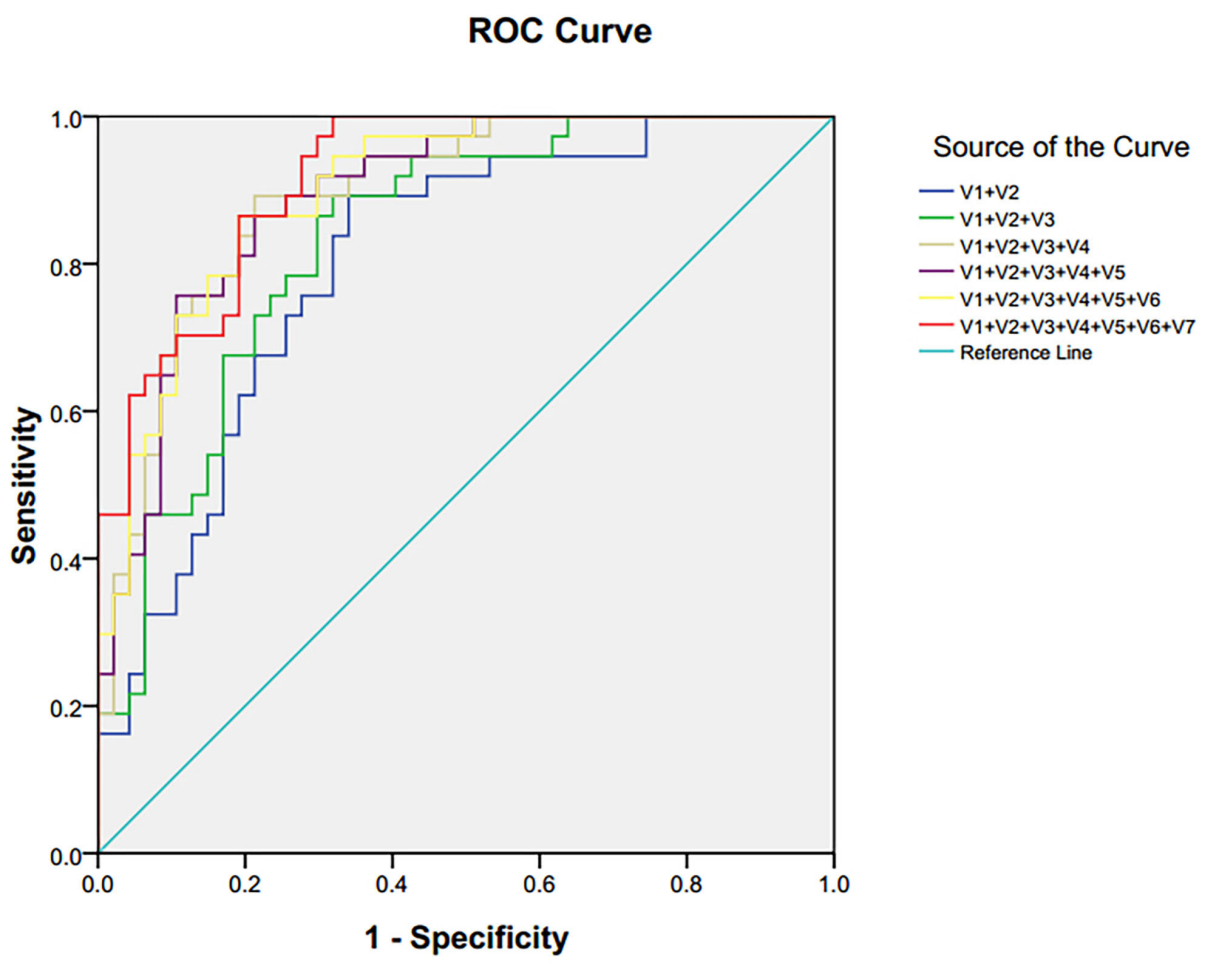
ROC curve of differential metabolites in plasma of patients with bipolar depression. V1 lactate, V2 trimethylamine oxide, V3 N-acetyl glycoprotein, V4α-glucose, V5 choline, V6β-glucose, V7 glycine.

Seven single biomarkers with low diagnostic performance were used as independent variables and combined freely to establish binary logistic; bipolar depression was the dependent variable. The predictive probability (P) was preserved as a combined diagnostic index. The ROC was established again (Figure 5). The combined diagnostic performance of four biomarkers (lactate, trimethylamine oxide, N-acetyl glycoprotein, and α-glucose) was higher in all combinations (AUC = 0.893; 95% confidence interval: 0.826–0.960) (Table 3). The specificity and sensitivity of these biomarkers were the greatest in all combinations, and this combined diagnostic function was higher than a single index. Therefore, the combination of four biomarkers in plasma—lactate, trimethylamine oxide, N-acetyl glycoprotein, and α-glucose—can be used as a biomarker group for the diagnosis of bipolar depression.

**Table 3:** AUC of differential metabolites in plasma of patients with bipolar depression.

**Metabolites**	**Area**	**Std. error^a^**	**Asymptotic sig.^b^**	**Asymptotic 95% confidence interval**	
				**Lower bound**	**Upper bound**
V1+V2	0.802	0.048	0.000	0.709	0.896
V1+V2+V3	0.835	0.043	0.000	0.750	0.920
V1+V2+V3+V4	0.893	0.034	0.000	0.826	0.960
V1+V2+V3+V4+V5	0.892	0.034	0.000	0.826	0.959
V1+V2+V3+V4+V5+V6	0.901	0.032	0.000	0.838	0.964
V1+V2+V3+V4+V5+V6+V7	0.917	0.028	0.000	0.862	0.972

a *Under the non-parametric assumption*.

b *Null hypothesis: true area = 0.5*.

*V1 lactate, V2 trimethylamine oxide, V3 N-acetyl glycoprotein, V4 α-glucose, V5 choline, V6 β-glucose, V7 glycine*.

## Discussion

At present, little is known about the metabolic mechanisms and appropriate objective diagnostic methods during the course of BD; this leads to a low diagnostic accuracy of this disorder. There is therefore an urgent need for a sensitive, specific, and accurate diagnostic method to be found and applied to the clinical diagnosis of the disorder. In this study, ^1^H NMR metabonomics was used to explore differential metabolites for the diagnosis of bipolar depression. We identified a panel consisting of seven potential biomarkers (lactate, trimethylamine oxide, N-acetyl glycoprotein, choline, α-glucose, glycine, and β-glucose). After ROC curve evaluation, it was found that the diagnostic ability of a single metabolite was poor. The reason may be the complex interaction between metabolites within the organism. A single metabolite could not fully reflect the occurrence and development of BD, while a combination was an more effective reflection. A combination consisting of lactate, trimethylamine oxide, N-acetyl glycoprotein, and α-glucose as group biomarkers showed a better diagnostic performance for bipolar depression with an AUC of 0.893.

To further investigate the biological functions of differential metabolites, pathway analysis was conducted using the online software MetaboAnalyst. It was found that potential metabolites were related to seven metabolic pathways. Among them, glycine, serine and threonine metabolism, and pyruvate metabolism showed a high correlation with bipolar depression. Maes et al. found significant changes of glycine, serine, and threonine levels in patients with treatment-resistant depression (28). Previous studies (20) showed that the serum level of serine (including L-serine and D-serine) in BD was significantly reduced, while the two enantiomers of serine in patients with Major Depressive Disorder (29) were higher than those in healthy control participants. In addition, the level of L-serine in patients with schizophrenia (30, 31) was increased, and the level of D-serine was decreased. Therefore, these serine enantiomers (D-serine and L-serine) may be implicated in the onset of psychiatric disorders.

Glucose is the main source of energy, while lactate is the final product of glucose metabolism under anaerobic conditions (32). In this research, the level of lactate in BD was significantly increased relative to healthy control participants, while α-glucose was decreased, indicating that energy metabolism might be disturbed in patients with bipolar depression. Consistent with our findings, previous studies have also observed elevated levels of lactate in post-mortem brain tissue obtained from patients with BD (14, 33, 34). Yoshimi et al. found abnormalities of the citric acid cycle in serum and cerebrospinal fluid obtained from male patients with BD (13, 20). These findings suggest that an energy metabolism disorder plays a significant role in the onset of BD. In addition, energy deficiency might relate to most common depressive symptoms, such as decreased activity, physical fatigue, and slowed cognitive function.

Trimethylamine oxide (TMAO) is one of the significant metabolites of gut microbiota. Nutrients rich in phosphatidylcholine and L-carnitine initially decompose gas Trimethylamine by the intestinal microbes, then the gas is oxidized by flavin monooxygenase 3 to form TMAO in the liver (35). In this study, the level of TMAO was decreased in BD subjects, indicating the dysbiosis of gut microbiota in patients with bipolar depression. Some studies found that there is a biphasic signal transduction system in the brain and gut microbiota, which is named the microbiota-gut-brain axis (36). Gut microbiota could affect brain function and emotional state by this axis (37). A recent review showed that the disturbed gut microbiota might implicated in many neuropsychiatric disorders, such as epilepsy, Alzheimer's disease, depression, and schizophrenia (38). Accumulating evidence suggested that the diversity of gut microbiota in BD was decreased (39–41). Meanwhile, a metabonomic analysis of urine demonstrated the relationship between gut microbiota and depressive-like behaviors (42). These findings highlight the significant role of gut microbiota in the onset of BD.

Metabonomics can detect and quantify overall metabolite composition in biological systems (5, 43), and the technique has already been used to provide information on disease processes, drug toxicity/therapy, gene function, etc. (44). However, the specific role of altered metabolites in disease occurrence and drug treatment mechanisms has not yet been understood. Clearly, the relationship may be much more complex than we have demonstrated, and further research is indicated.

The results of this research should be considered in the light of several limitations. First, a relatively small sample size of BD subjects was recruited. Second, the cross-sectional research only reveals the correlation between biomarkers and diseases, causal link still needs longitudinal research to explore. Third, this study does not consider the impact of psychotropic substance on metabolites, which may be confounding factors affecting the results. In the future, we will expand the sample to further explore the differences in metabolic pathways between patients taking and not taking drugs. Finally, this research only selects patients with depressive episodes of bipolar disorder. In the future, we will compare the different episodes of the disease and explore the dynamic trajectory of metabolomics changes in bipolar patients.

Using an NMR-based metabonomic approach, we identified a panel of four potential biomarkers (lactate, trimethylamine oxide, N-acetyl glycoprotein, and α-glucose) which revealed a good combined capacity for diagnosing BD patients during a depressive episode. The analysis of metabolic pathways suggests that disturbances of energy and amino acid metabolisms might be involved in the pathogenesis of bipolar depression.

## Data Availability Statement

The raw data supporting the conclusions of this article will be made available by the authors, without undue reservation.

## Ethics Statement

The studies involving human participants were reviewed and approved by the Medical Ethics Committee of Shanxi Bethune Hospital. All subjects and their guardians voluntarily participated in the study and gave informed consent. For the teenage participants, informed consent was signed by their parents. Experienced and licensed psychiatrists participated in the recruiting procedure.

## Author Contributions

HY and YR designed the study. SB, YR, and X-lS wrote the manuscript. J-ST and YJ performed the experiment. Subject recruitment was performed by X-lS, X-xC, and X-yB. All authors contributed to the article and approved the submitted version.

## Conflict of Interest

The authors declare that the research was conducted in the absence of any commercial or financial relationships that could be construed as a potential conflict of interest.
